# Optimization-based design of a heat flux concentrator

**DOI:** 10.1038/srep40591

**Published:** 2017-01-13

**Authors:** Ignacio Peralta, Víctor D. Fachinotti, Ángel A. Ciarbonetti

**Affiliations:** 1 Centro de Investigación de Métodos Computacionales (CIMEC), Universidad Nacional del Litoral (UNL)/Consejo Nacional de Investigaciones Científicas y Técnicas (CONICET), Predio CONICET Santa Fe “Dr. Alberto Cassano”, Colectora Ruta Nac Nro 168, Km 0, Paraje El Pozo, 3000 Santa Fe, Argentina

## Abstract

To gain control over the diffusive heat flux in a given domain, one needs to engineer a thermal metamaterial with a specific distribution of the generally anisotropic thermal conductivity throughout the domain. Until now, the appropriate conductivity distribution was usually determined using transformation thermodynamics. By this way, only a few particular cases of heat flux control in simple domains having simple boundary conditions were studied. Thermal metamaterials based on optimization algorithm provides superior properties compared to those using the previous methods. As a more general approach, we propose to define the heat control problem as an optimization problem where we minimize the error in guiding the heat flux in a given way, taking as design variables the parameters that define the variable microstructure of the metamaterial. In the present study we numerically demonstrate the ability to manipulate heat flux by designing a device to concentrate the thermal energy to its center without disturbing the temperature profile outside it.

The control of the electromagnetic flux using metamaterials led to major innovations in electronics and communications^1^. Taking advantage of the analogies between electromagnetism and thermodynamics, some researchers developed materials with unprecedented thermal properties (the thermal “metamaterials”) for heat flux manipulation, for instance the heat flux inverter by Narayana and Sato^2^.

Compared to the advances in electromagnetism, the design of thermal metamaterials is an emerging research and development area. In a first approach, metamaterials can be empirically designed (e.g., the thermal shield of Narayana and Sato^2^). More sophisticated thermal metamaterials can be designed using the transformation thermodynamics concept (e.g., the inverter and the concentrator proposed by Narayana and Sato^2^ or the cloaking device of Schittny *et al*.^3^, inherited from electromagnetism^4^). A straightforward example of the application of ideas from electromagnetism in thermal problems is the heat flux inverter of Narayana and Sato^2^, derived from the device to rotate electromagnetic fields proposed by Chen and Chan^5^ to rotate electromagnetic fields.

The transformation-based approach has been applied to specific heat control problems. For general problems (i.e., having arbitrary prescribed magnitude and direction of the heat flux, geometry of the manipulating device, geometry and boundary conditions of the domain where the device is embedded) we propose a new, optimization-based approach for the design of thermal metamaterials. A variety of optimization algorithms have been used to design efficient metamaterials but only in the field of photonic^6^,^7^,^8^,^9^,^10^. Here we solve a nonlinear constrained optimization problem where the objective function to minimize is the error in the accomplishment of the given heat manipulation task, and the design variables characterize the spatial distribution of the metamaterial throughout the manipulating device.

We show the capability of the present method by designing a device for thermal concentration, as alternative to the transformation-based design of Chen and Lei^11^, using an interior point optimization algorithm.

## Definition of the heat flux guidance problem

**Figure 1:**
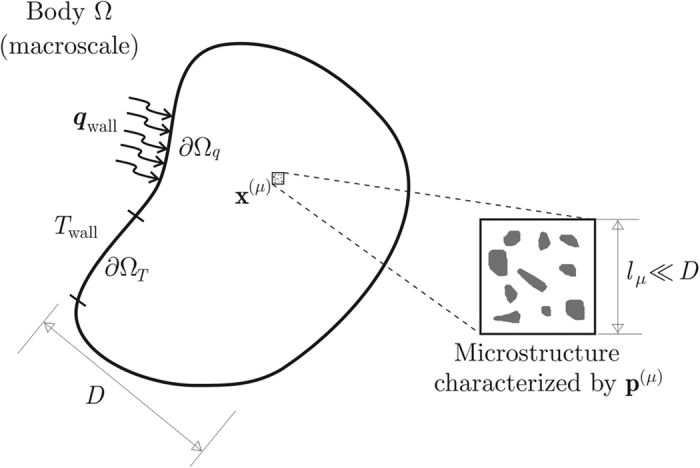
Thermal problem in a macroscopic domain Ω where the effective properties depend on a quantitatively characterized microstructure.

Let us consider the domain Ω in Fig. 1, with boundary ∂Ω divided in two non-overlapping portions: ∂Ω_*q*_ (where the heat flux *q*_wall_ is prescribed) and ∂Ω_*T*_ (where the temperature *T*_wall_ is prescribed). In steady state, the heat flux conduction in Ω is governed by the equation


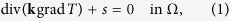


and the boundary conditions:


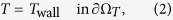



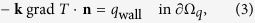


where *T* is the temperature, *s* is the internal heat source, **k** is the (effective) thermal conductivity tensor, and **n** is the unit vector normal to and pointing outwards ∂Ω.

Using the finite element method (FEM), the temperature field in Ω is approximated as follows:


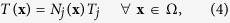


where *N*_*j*_ is the shape function associated to the node *j* of the finite elements mesh (discretized Ω) and *T*_*j*_ is the temperature at node *j* (unknown). Using a standard (Galerkin) FEM, the nodal temperature *T*_*j*_ is the solution of the algebraic system of equations


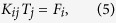


where *K*_*ij*_ and *F*_*i*_ are the components of the global conductivity matrix and the nodal heat flux vector respectively, given by


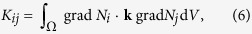



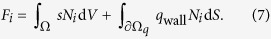


The system of equations (5) is the FEM version of the heat conduction (1) subject to the boundary conditions (2) and (3). This is a classical FEM problem, whose solution has been extensively detailed in classical FEM literature, for instance in the book of Zienkiewicz and Taylor on the basics of FEM^12^.

### Influence of the inhomogeneous microstructure on the macroscopic thermal response

Let the microstructure vary throughout Ω and be sampled at a series of points **x**^(*μ*)^ ∈ Ω (*μ* = 1, …, *N*). Further, let the microstructure at any **x**^(*μ*)^ be characterized by *n* parameters 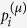, grouped in the vector **p**^(*μ*)^. Then, the effective conductivity **k** at **x**^(*μ*)^ is at last a function of **p**^(*μ*)^, i.e.


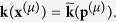


The microstructure throughout Ω is characterized by the vector **P** = [**p**^(1)^, …, **p**^(*N*)^]. Then, the global conductivity matrix **K** (whose components are given by equation (6)) is a function of **P**, and so they are the nodal temperatures *T*_*j*_ (solution of equation (5)) and the temperature field *T* (approximated by equation (4) for FEM).

### Task accomplishment as an optimization problem

**Figure 2:**
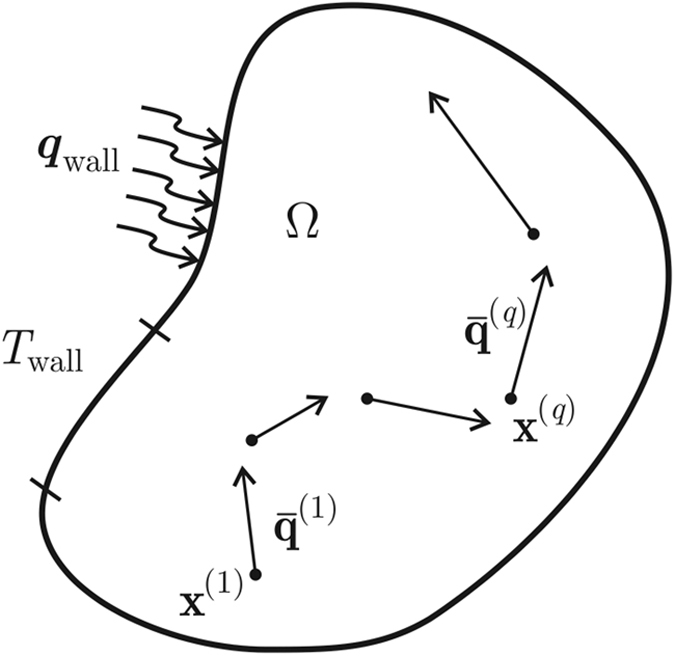
The heat flux guidance problem in the macroscopic domain Ω; 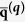 denotes the desired heat flux at the point x^(q)^ ∈ Ω.

To design the microstructure in the macroscopic domain Ω consists of finding **P** such that Ω responds in a desired way. In this case, we aim to enforce the heat flux to take the magnitude as well as the direction of the vector 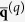 at a series of points **x**^(*q*)^ ∈ Ω, *q* = 1, …, *Q*, as shown in Fig. 2. The heat flux at any **x**^(*q*)^ is given by


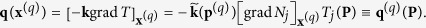


Then, we have to find **P** such that


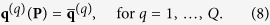


Let us look for **P** within a space 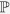 of admissible solutions. Generally, the task (8) cannot be exactly satisfied by any 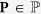. So, let us accomplish this task as well as possible by solving the nonlinear constrained optimization problem


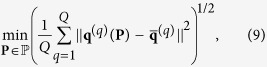


where the objective function is the root mean square (RMS) error in the accomplishment of the task (8).

## Application to a heat concentration and cloaking problem

Let us apply the proposed optimization-based approach to design a device for heat concentration as alternative to that designed by Chen and Lei^11^ based on transformation thermodynamics. This device, embedded in a plate with prescribed heat flux, is designed to concentrate the thermal energy at its center while keeping the outer flux unaltered (i.e., cloaking the device).

**Figure 3:**
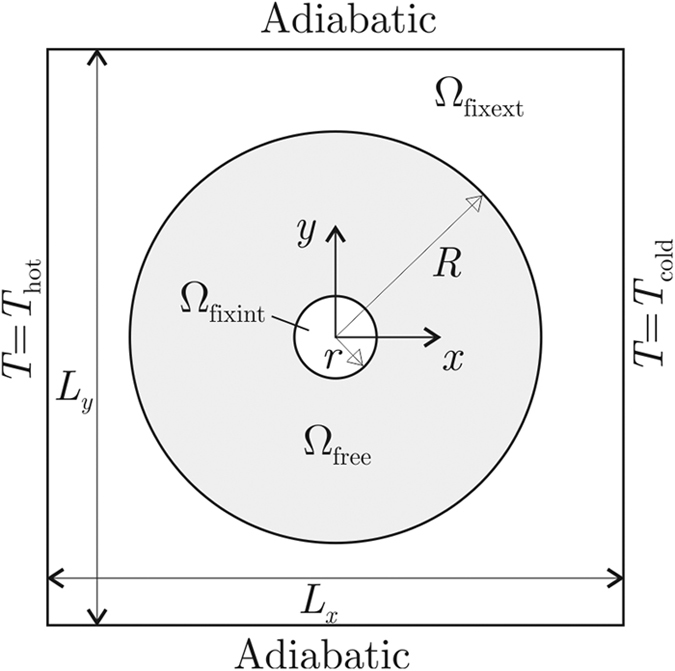
Domain of analysis for the heat concentration and cloaking problem.

The Ω domain is the entire plate, a square of sides *L*_*x*_ = *L*_*y*_ = 14 cm subject to the following boundary conditions: *T* = *T*_max_ = 100 °C for *x* = −*L*_*x*_/2, *T* = *T*_min_ = 0 °C for *x* = *L*_*x*_/2, and **q** ⋅ **n** = 0 for *y* = ±*L*_*y*_/2 (see Fig. 3). The heat flux normal to the plate is neglected. The plate is made of 40%-nickel steel with homogeneous and isotropic thermal conductivity *k*_ns_ = 10 W/(mK). Without the device, the heat flux in the plate is


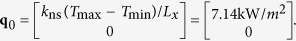


The considered device is the ring Ω_free_ ⊂ Ω with inner and outer radii *r* = 1 cm and *R* = 5 cm, see Fig. 3. This ring is designed to thermally concentrate the heat flux in the region Ω_fixint_. A further design requirement on the device is to keep the heat flux outside it (i.e., in the remainder portion of the plate, Ω_fixext_) unaltered.

**Figure 4:**
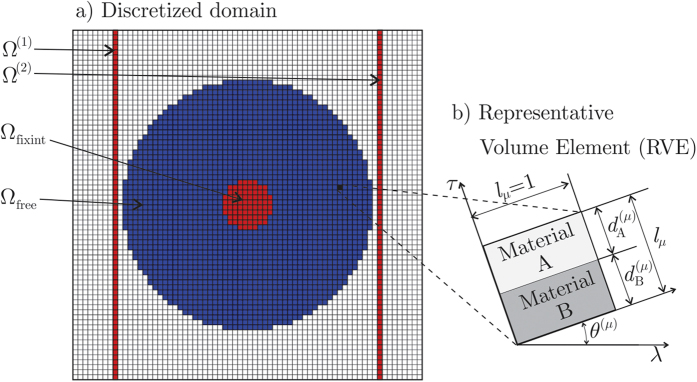
(**a**) Finite element mesh of the analyzed domain Ω; the blue elements belong to the device, and the red ones have prescribed heat flux. (**b**) Representative volume element (RVE) of the microstructure at a point **x**^(*μ*)^ in the the heat flux manipulating device Ω_free_.

The domain Ω is discretized using a mesh of 70 × 70 bilinear rectangular finite elements, as shown in Fig. 4(a). Each blue element, belonging to the device Ω_free_, has a microstructure sampling point. In the other elements, the material is nickel steel.

Regarding the mesh refinement, it is well known that it affects the optimal result, as it is widely discussed in the book of Bendsøe and Sigmund^13^. Normally, the finer the mesh, the more optimal the solution. The current choice was found to be a good deal between optimality and computational cost.

### Definition of the metamaterial for anisotropic heat conduction

Following Narayana and Sato^2^, the anisotropy in the effective conductivity of the device Ω_free_ is achieved by using a stacked composite or laminate made of alternating sheets of materials A and B with different isotropic conductivities. As pointed out by Schittny *et al*.^14^, a laminate is a metamaterial because its effective conductivity, being anisotropic, goes beyond the conductivities of its constituents, which are isotropic.

Like Chen and Lei^11^, we adopted A = copper and B = polydimethylsiloxane (PDMS), with isotropic conductivities *k*_copper_ = 398 W/(mK) and *k*_PDMS_ = 0.27 W/(mK). The use of laminates of materials with markedly distinct conductivity at the microstructural level leads to a highly anisotropic effective conductivity, which is a key issue for guiding the heat flux. Actually, it is a popular choice in the literature^2^,^11^,^15^,^16^.

As shown in Fig. 4(b), the representative volume element (RVE) of the microstructure of such composite at the sampling point **x**^(*μ*)^ ∈ Ω_free_ is a unit square characterized by the vector **p**^(*μ*)^ of components 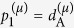 (thickness of sheet of material A) and 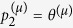 (orientation of the sheets); the thickness of the sheet of material B is *d*_B_ = *l*_*μ*_ − *d*_A_, where *l*_*μ*_ = 1 is the thickness of the RVE. The effective thermal conductivities at **x**^(*μ*)^ in the direction of the sheets (*λ*) and normal to the sheets (*τ*) are^17^


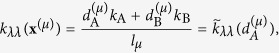



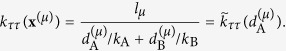


These are principal conductivities, to be arranged in the matrix


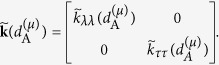


Now, the matrix of tensorial components of the effective conductivity referred to the fixed Cartesian frame *x*-*y* at the point **x**^(*μ*)^ can be computed as


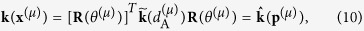


where **R** is the rotation matrix


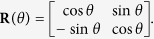


The equation (10) explicitly defines the effective conductivity at a point as a function of the microstructure at that point.

### Optimization settings

To design the current device implies to solve the optimization problem given by equation (9). The cloaking task is prescribed by setting 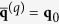 at the center of the elements in Ω^(1)^ and Ω^(2)^, while the heat concentration task is forced by setting 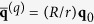 at the center of elements in Ω_fixint_, with *R*/*r* = 5 in this case. Note that the vector **P** contains as variables only the vectors **p**^(*μ*)^ characterizing the microstructure at the *N* = 1896 elements of Ω_free_, with 
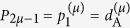
 and 
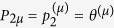
, *μ* = 1, 2, … *N*.

For the chosen metamaterial, the current optimization problem is subject to the following box constraints:


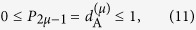



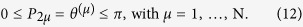


Here, this nonlinear constrained optimization problem was solved using the IPOPT interior point algorithm^18^. Additional constraints may serve to avoid “complications”^13^: dependence on the finite element mesh, numerical instabilities, non-uniqueness of the solution, presence of multiple minima, etc. For the purpose of the current work, only the above box constraints are considered.

## Results

**Figure 5:**
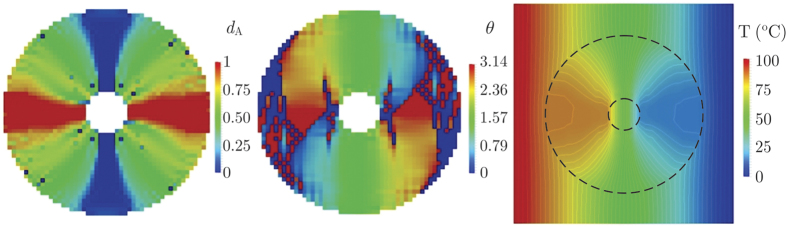
Optimal solutions without density filtering for the distribution of the fraction of copper (*d*_A_), the orientation of copper and PDMS sheets (*θ*), and the temperature *T* in the concentrator device.

The optimal solutions for *d*_A_ (that is in fact the fraction of copper since the RVE was assumed to be a unit square) and *θ* (the orientation of the sheets) in the device are plotted in Fig. 5, together with the corresponding temperature distribution. Note that the device accomplished the combined task up to an RMS error equal to = 1.67 kW/m^2^ = 0.23||**q**_0_||.

Although we considered this error to be small enough, the solution is seriously affected by “checkerboard”-type instabilities, mainly in the orientation field (Fig. 5 at the center). This is a well-known and widely studied defect in material distribution problems (see the book of Bendsøe and Sigmund^13^ and references therein), which can be avoided using the density filter technique proposed by Sigmund^19^. The components of the vector **P** are still the design variables for the optimization problem (8), but the objective function in equation (9) as well as the constraints (11) and (12) are evaluated for the vector of physical or filtered parameters 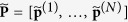. Then, the microstructure at the finite element *e* in Ω_free_ is actually characterized by the vector 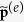, which is defined as


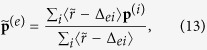


where 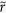 is the filter radius (to be adopted), measured from the center of the finite element *e*, **p**^(*i*)^ is the vector of design variables associated to the finite element *i*, Δ_*ei*_ is the distance between the centers of the elements *e* and *i*, and 〈*x*〉 is the ramp function (〈*x*〉 = *x* for *x* > 0, and 〈*x*〉 = 0 for *x* ≤ 0).

**Figure 6:**
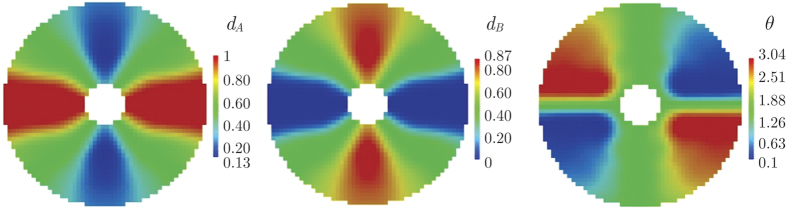
Optimal solutions using density filtering for the distribution of the fraction of copper (*d*_A_), the fraction of PDMS (*d*_B_), and the orientation of copper and PDMS sheets in the concentrator device.

Now, solving the optimization problem (9) using density filtering with 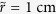 (i.e., five times the finite element size), we obtain the metamaterial distribution shown in Fig. 6, which is completely checkerboard-free. The so-obtained device Ω_free_ has a crosslike structure, with horizontal arms (parallel to **q**_0_) mostly made of highly-conductive copper and vertical arms (normal to **q**_0_) mostly made of poorly-conductive PDMS.

**Figure 7:**
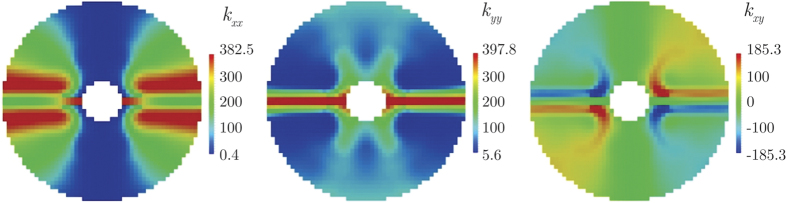
Distribution of the Cartesian components of the effective thermal conductivity in the concentrator device, given in W/(mK).

**Figure 8:**
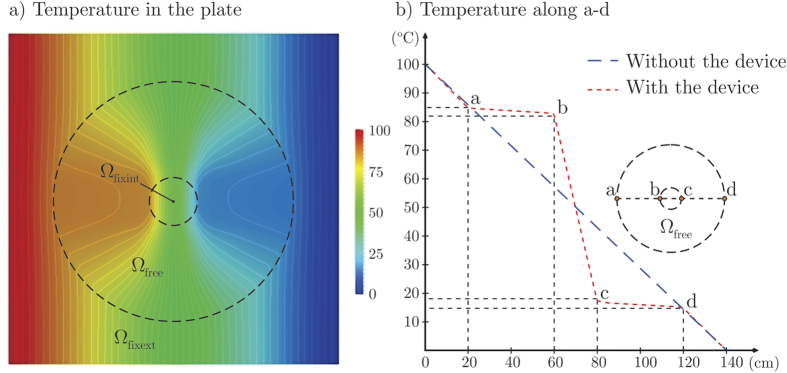
(**a**) Temperature distribution in the plate, in °C; the difference between isotherms is 2 °C. (**b**) Temperature profile along (a–d).

As consequence of the metamaterial distribution depicted in Fig. 6, the effective thermal conductivity varies inside the device as shown in Fig. 7, being generally anisotropic. Thanks to this conductivity distribution in the device, the temperature field in the whole plate is that shown in Fig. 8(a). There, it can be qualitatively realized how well the given combined cloaking and concentration task was accomplished: First, isotherms are almost parallel and equally spaced outside the device, as it would be the case without the device. Secondly, inside the device, the isotherms are significantly bent towards the inner region (Ω_fixint_), clearly demonstrating the strong energy concentration. This could be quantitatively appreciated in Fig. 8(b): The temperature along the center line *y* = 0, that where the heat flux is the most altered by the device, is slightly modified outside the device, while it has a gradient 4.58 times greater than the original one in the center of the device. Let us remark that, as solution of the optimization problem, the combined cloaking and concentration task was accomplished up to an RMS error equal to 2.04 kW/m^2^ = 0.29 ||**q**_0_||; individually, the RMS error for the concentration task in Ω_fixint_ was 3.34 kW/m^2^ = 0.09 (*R*/*r*)||**q**_0_||, while the RMS error for the cloaking task in Ω^(1)^ and Ω^(2)^ was equal to 0.39 kW/m^2^ = 0.05 ||**q**_0_||. Although the minimization problem (9) accounts only for the portions Ω^(1)^ and Ω^(2)^ of Ω_fixext_, the heat flux is practically unaltered all around Ω_fixext_: it approaches **q**_0_ with a RMS error equal to 0.07 ||**q**_0_||.

Chen and Lei^11^ defined a concentration efficiency index as *f* = |(*T*_b_ − *T*_c_)/(*T*_a_ − *T*_d_)|, where *b* and *c* are points located in the boundary of the heat concentration region Ω_fixint_, and *a* and *d* are points located at the outer boundary of the device Ω_free_ (see Fig. 8(b)). For the current device, we obtain *f* = 94.2%, close to the ideal *f* = 100%. Let us recall that the device designed by Chen and Lei based on the transformation approach, made of 100 radial copper-PDMS laminates, had a theoretical efficiency *f* = 96.3%, which fell to *f* = 88.1% for the finally fabricated device.

Comparing the current device to Chen and Lei’s one, it appears a crucial advantage of the current optimization-based design with respect to the transformation-based design: the device is designed just for the desired task (to manipulate an originally one-direction heat flux), avoiding to “oversize” it by performing unwanted or unprescribed tasks (in the case of Chen and Lei’s device, to manipulate the heat flux coming from any direction). Further, if the task is only to concentrate heat, the cloaking task is just a collateral result of applying the transformation-based approach.

Note that the current device can be seen as a neutral inclusion because of its cloaking effect. A priori, according to the theory of neutral inclusions (see chapter 7 in the book of Milton^20^ and references therein), it would be possible to determine in an analytical way a homogeneous isotropic material to make the cloaking device, a solution that is considerably more convenient than the current one using a heterogeneous metamaterial. However, let us remark that this trivial solution is not applicable to the current case. Actually, applying the Hashin-Shtrikman formula^21^ with the regions outside and enclosed by the device having the same isotropic conductivity *k*_ns_, we determine that the only way of keeping the exterior flux unaltered is choosing the isotropic conductivity inside the device also equal to *k*_ns_, which leads to completely ignore the heat flux concentration task.

## Conclusions

We presented a novel method for designing metamaterials to control the diffusive heat flux in ways that were inconceivable using ordinary materials. This method consists in solving an optimization problem where the objective function to be minimized is the error in the accomplishment of a given heat flux control task, and the design variables define the microstructure in a heat flux manipulating device. Its potentiality was proved by designing a device for energy concentration that has close-to-ideal efficiency and, at the same time, leaves the external heat flux practically unaltered.

We expect these results may create opportunities to develop new advanced engineered materials for enhancing the efficiency of thermal devices in solar thermal collectors, for instance.

Future work will be devoted to ensure the manufacturability of these optimization-based designs.

## Additional Information

**How to cite this article**: Peralta, I. *et al*. Optimization-based design of a heat flux concentrator. *Sci. Rep.* **7**, 40591; doi: 10.1038/srep40591 (2017).

**Publisher's note:** Springer Nature remains neutral with regard to jurisdictional claims in published maps and institutional affiliations.
